# Antibiofilm Effect of *Lavandula multifida* Essential Oil: A New Approach for Chronic Infections

**DOI:** 10.3390/pharmaceutics15082142

**Published:** 2023-08-15

**Authors:** Jorge Alves-Silva, Mónica Zuzarte, Carlos Cavaleiro, Lígia Salgueiro

**Affiliations:** 1 Univ Coimbra, Faculty of Pharmacy, Azinhaga de S. Comba, 3000-548 Coimbra, Portugal; jmasilva@student.ff.uc.pt (J.A.-S.); cavaleir@ff.uc.pt (C.C.); 2 Univ Coimbra, Coimbra Institute for Clinical and Biomedical Research (iCBR), Faculty of Medicine, Azinhaga de S. Comba, 3000-548 Coimbra, Portugal; 3 Univ Coimbra, Center for Innovative Biomedicine and Biotechnology (CIBB), 3000-548 Coimbra, Portugal; 4 Clinical Academic Centre of Coimbra (CACC), 3004-561 Coimbra, Portugal; 5 Univ Coimbra, Chemical Process Engineering and Forest Products Research Centre (CIEPQPF), Department of Chemical Engineering, Faculty of Sciences and Technology, Rua Sílvio Lima, 3030-790 Coimbra, Portugal

**Keywords:** antifungal, candidiasis, dermatophytosis, lavender, virulence

## Abstract

Fungal infections are associated with high morbidity and mortality rates, being highly prevalent in patients with underlying health complications such as chronic lung disease, HIV, cancer, and diabetes mellitus. To mitigate these infections, the development of effective antifungals is imperative, with plants standing out as promising sources of bioactive compounds. In the present study, we focus on the antibiofilm potential of *Lavandula multifida* essential oil (EO) against dermatophyte strains and *Candida albicans*. The EO was characterized using GC and GC–MS, and its antifungal effect was assessed on both biofilm formation and disruption. Biofilm mass, extracellular matrix, and viability were quantified using crystal violet, safranin, and XTT assays, respectively, and morphological alterations were confirmed using optical and scanning electron microscopy. *L. multifida* EO showed very high amounts of carvacrol and was very effective in inhibiting and disrupting fungal biofilms. The EO significantly decreased biofilm mass and viability in all tested fungi. In addition, a reduction in dermatophytes’ extracellular matrix was observed, particularly during biofilm formation. Morphological alterations were evident in mature biofilms, with a clear decrease in hypha diameter. These promising results support the use of *L. multifida* EO in the development of effective plant-based antifungal products.

## 1. Introduction

Fungal infections, or mycosis, represent a huge health and economic burden affecting over 1 billion individuals worldwide and accounting for around 13 million infections per year [1]. Indeed, the Global Action Fund for Fungal Infections (GAFFI) estimates that over 300 million individuals are affected by a severe fungal infection every year [2]. Moreover, patients with underlying health concerns or a weakened immune system, including chronic lung disease, prior tuberculosis, HIV, cancer, and diabetes mellitus, are at higher risk [3]. In addition, in patients with HIV, the relapse rates of onychomycosis are higher than in healthy individuals [4], reinforcing the relevance of these infections in debilitated patients.

The main etiologic agents of superficial fungal infections belong to *Epidermophyton*, *Microsporum,* and *Trichophyton* filamentous fungi genera that affect the skin, nails, and hair [5]. These infections, collectively named dermatophytosis, although not lethal, are the most disseminated type of mycosis, affecting between 20 and 25% of the world’s population [6]. Dermatophytosis is generally neglected but is a major cause of morbidity-associated superficial mycoses, with frequent relapses and high resistance to therapy [7]. Indeed, therapy duration is usually very long, leading to poor patient adherence and consequent infection relapses. Moreover, these fungi are known to produce biofilms [8,9], thus further contributing to their resistance to antifungal therapy and the consequent persistence of infections.

On the other hand, invasive infections, although less prevalent than superficial mycosis, account for higher mortality rates, being responsible for 1.5 million annual deaths [1], with candidiasis being the most predominant type [10]. Despite the emergence of non-*albicans Candida* infections, *Candida albicans* remains the most widespread strain in humans [11]. An overgrowth of this fungi causes candidiasis with vaginal yeast infection, diaper rash, and thrush being the most common types of infection. More severe cases called invasive candidiasis target the whole body, including the blood, bones, brain, and heart. Similarly, to dermatophytes, *C. albicans* also has the capacity to form biofilms that pose a threat to the individual, as these structures are very resistant to treatment and lead to chronic and persistent infections [12,13,14].

Both dermatophytosis and candidiasis constitute a very serious health concern that requires effective therapeutic strategies. Despite the recent developments in antifungals with the increasing use of echinocandins and third-generation azoles, invasive fungal infections continue to impact patients’ quality of life, and mortality rates remain high. These poor outcomes are usually associated with antifungals’ high toxicity, unpleasant side effects, drug interactions, and, most importantly, the emergence of resistant strains [15,16]. Indeed, the extensive use of non-selective antifungals has contributed to the emergence of fungi resistance with associated off-target toxicity and treatment failure. Global warming is also thought to lead to the emergence of new fungal diseases [17] as the gap between environmental and host temperatures narrows [18] and the fungi seem to easily adapt [17], thus pushing the burden of these infections.

Aromatic plants represent a valuable source of antifungal compounds, and particularly those from the genus *Lavandula* have shown very promising antifungal properties [19,20,21,22,23,24,25,26,27]. In a previous study, we reported the antifungal potential of fernleaf lavender (*Lavandula multifida*) against several pathogenic fungi and pointed out a possible mechanism of action through initial metabolic arrest followed by cell death in *C. albicans* [27]. In the present study, we intend to deepen our knowledge by exploring the effect of this essential oil on dermatophytes and *C. albicans* biofilms. These highly organized complex microbial communities, involved by self-produced extracellular matrix, are known to protect the fungi from the surrounding environment and constitute reservoirs for persistent infections [28]. In addition, the fungi growing within biofilms present phenotypic alterations that promote resistance to antifungal drugs compared to planktonic cells [29,30,31]. Indeed, biofilms are very difficult to eradicate due to their high resistance to conventional therapy and host defenses [32]. Therefore, targeting these virulence factors is of utmost importance and represents a very attractive strategy for the development of effective antifungal drugs.

In the present study, we highlight the antibiofilm potential of *L. multifida* essential oil against several strains of dermatophytes and *C. albicans*. Our studies point out promising results for the majority of the strains tested, with *Epidermophyton floccosum* being the most susceptible. Overall, the antivirulence potential of *L. multifida* essential oil, together with its inhibitory effect on fungal growth, as previously reported, justifies the development of effective essential oil-based antifungal products.

## 2. Materials and Methods

### 2.1. Plant Material and Essential Oil Characterization

*Lavandula multifida* essential oil was obtained using hydrodistillation for 3 h from fresh flowering aerial parts of plants collected in Sesimbra region, Portugal. Plant authenticity was verified by the taxonomist Dr. Jorge Paiva (University of Coimbra). Voucher specimens were deposited at the Herbarium of the Department of Life Sciences of the University of Coimbra, Portugal. The essential oil was characterized using gas-chromatography (GC, Agilent Technologies, Palo Alto, CA, USA) coupled with mass spectrometry (GC–MS, Agilent Technologies), as previously described [33].

### 2.2. Antifungal Effect of L. multifida Essential Oil

#### 2.2.1. Fungal Strains

The antibiofilm potential of *L. multifida* essential oil was assessed on yeasts and filamentous fungi. Dermatophyte species included both collection strains such as *Microsporum gypseum* CECT 2908, *Trichophyton mentagrophytes* var. *interdigitale* CECT 2958, and *T. rubrum* CECT 2794, and clinical strains, namely *Epidermophytom floccosum* FF9, *M. canis* FF1, and *T. mentagrophytes* FF7, whereas *C. albicans* ATCC 10231 was the representative yeast strain tested. Strains were cultured in potato dextrose agar (Oxoid Limited, Hampshire, UK) or Sabouraud agar (Oxoid Limited) at 37 °C for 48 h (*C. albicans*) or 30 °C for 7 days (dermatophytes).

#### 2.2.2. Fungal Inoculum

Dermatophyte inoculums were prepared from 7-day-old cultures in Sabouraud agar by adding sterile 0.9% NaCl and vigorously vortexed to detach conidia. The saline was then transferred to a new sterile tube and left to settle for 5 min to allow the separation of the conidia from hyphae. The supernatant was collected to a sterile tube and turbidity was adjusted to 1 unit of McFarland scale, containing approximately 1 × 10^6^ conidia/mL.

*C. albicans* was grown in a yeast peptone dextrose (YPD) medium (1% yeast extract, 2% peptone, and 2% dextrose, Oxoid Limited) inoculated from 24 h old cultures in Sabouraud agar for 24 h at 37 °C. Afterward, the medium was removed, and yeasts were washed twice with PBS (0.8% NaCl, 0.02% KH_2_PO_4_. 0.31% Na_2_HPO_4_.12 H_2_O and 0.02% KCl; pH 7.4), with centrifugations of 3000× *g* for 10 min between each washing step. Cell density was adjusted to 1 × 10^6^ CFU/mL in RPMI 1640 pH 7.0 (with L-glutamine, without bicarbonate, Sigma-Aldrich, Burlington, MA, USA) supplemented with MOPS (Sigma-Aldrich).

#### 2.2.3. Effect of *L. multifida* Essential Oil on Biofilm Formation

To assess the effect of *L. multifida* essential oil on biofilm formation, the method described by Ali and colleagues was used [34], with some modifications. For dermatophytes, 200 µL of conidia suspension was added to sterile 96-well flat-bottom polystyrene microtiter plates and incubated at 37 °C for 3 h to allow for conidia to adhere. Then, saline was removed, and wells were washed with PBS (pH 7.4) to remove non-adherent cells. Afterward, 200 µL of RPMI medium containing different concentrations of the essential oil (0.32–0.04 µL/mL) was added and incubated for 72 h at 37 °C. Negative and positive controls containing non-inoculated medium and EO-free inoculated medium were included, respectively.

For *C. albicans*, 100 µL of the cell suspension was added to sterile 96-well flat-bottom polystyrene microtiter plates, and then 100 µL of RPMI medium containing different concentrations of the essential oil was added in order to achieve the final essential oil concentration ranging from 0.64 to 0.08 µL/mL. Plates were then incubated for 24 or 48 h. Negative and positive controls containing non-inoculated medium or EO-free inoculated medium were included, respectively.

#### 2.2.4. Effect of the *L. multifida* Essential Oil on Mature Biofilms

The capacity of the essential oil to disrupt mature biofilms was determined using the method reported by Ali and colleagues [34], with slight adaptations. Dermatophyte conidia suspensions (200 µL) were left to adhere for 3 h at 37 °C in sterile 96-well flat-bottom polystyrene microtiter plates. After saline removal, cells were washed with PBS to remove non-adherent cells, and 200 µL of sterile RPMI medium was added to the plates and left to incubate for 72 h at 37 °C. Then, the medium was removed, and following a washing step with PBS, 200 µL of RPMI with different concentrations of the essential oil (0.32–0.04 µL/mL) was added to the respective well and plates were then further incubated for 24 h at 37 °C. Negative and positive controls were considered as referred to in Section 2.2.3.

A cell suspension of *C. albicans* (100 µL) was added to a sterile 96-well flat-bottom polystyrene microtiter plate containing 100 µL of sterile RPMI medium and incubated for 24 h at 37 °C. Then, the medium was removed, and after a washing step with PBS, 200 µL of RPMI medium containing different concentrations of the essential oil (0.64–0.08 µL/mL) was added to the respective well and plates were then further incubated for 24 h at 37 °C. Negative and positive controls were considered as referred to in Section 2.2.3.

#### 2.2.5. Biofilm Mass

Crystal violet staining was carried out to quantify biofilm mass. Dermatophyte biofilms were stained according to the method reported by Castelo-Branco and colleagues [35], with slight modifications. Briefly, following medium removal, cells were washed with PBS to remove non-adherent cells. Then, biofilms were fixed with absolute methanol for 10 min. Afterward, 100 µL of 0.5% crystal violet solution was added and left to stain the biofilms for 15 min. Following crystal violet removal, biofilms were washed twice with sterile water. Crystals were dissolved using 150 µL of 33% acetic acid. The volume was transferred to new wells, and the absorbance was read at 620 nm.

*C. albicans* biofilms were stained using the procedure reported by Raut and colleagues [36]. Briefly, after medium removal, biofilms were air-dried and fixed with absolute methanol for 15 min. Then, plates were dried and 0.02% of crystal violet was added for 15 min. Excess solution was removed and wells were washed twice with sterile water, followed by the addition of 150 µL of 33% acetic acid to dissolve the crystals. The solution was transferred to new wells and the absorbance read at 620 nm.

Biomass reduction was calculated according to the following equation:

Biomass (%) = Abs treatment/Abs CT × 100

Abs CT and Abs treatment represent the absorbance at 620 nm for control and treated biofilms, respectively.

#### 2.2.6. Extracellular Matrix

Dermatophyte biofilm’s extracellular matrix was quantified using safranin red according to Costa-Orlandi and colleagues [37] with slight modifications. Following medium removal, biofilms were washed with PBS to remove non-adherent cells, and 100 µL of 0.5% safranin red solution was added and left to stain the biofilms for 5 min. Then, the solution was removed, and biofilms were washed twice with sterile PBS to remove the unbounded dye. Safranin crystals were released from the biofilm using 33% acetic acid and after transference to new wells, the absorbance was read at 520 nm. The reduction in biofilm extracellular matrix was calculated using the following equation:

Extracellular matrix (%) = Abs treatment/Abs CT × 100

Abs CT and Abs treatment represent the absorbance at 520 nm for control and treated biofilms, respectively.

#### 2.2.7. Biofilm Viability

The XTT (2.3-bis (2-methoxy-4-nitro-5-sulfophenyl)-5-[carbonyl (phenylamino)]-2H-tetrazolium hydroxide) reduction assay was used to determine the metabolic activity of biofilms, as previously reported [38]. After discarding the medium, biofilms were washed with PBS. Then, 100 µL of 1 mg/mL of XTT salt with 4 µM of menadione (from a solution of 10 mM made in acetone) was added, and biofilms were further incubated for 3 h at 37 °C. At the end of this period, the absorbance was read at 490 nm, and metabolic activity was measured using the following equation:

Metabolic activity (%) = Abs treatment/Abs CT × 100

Abs CT and Abs treatment represent the absorbance at 490 nm for control and treated biofilms, respectively.

#### 2.2.8. Biofilm Morphology and Ultrastructure

Morphological differences in biofilms in the presence of the essential oil were assessed using bright-field microscopy. Briefly, after XTT incubation, z-stacks of the biofilms were acquired using a bright-field microscope Ziess Axio XPD IRE 2 (Carl Zeiss, Oberkochen, Germany) equipped with a 40× objective LD Plan-Neofluar 40x/0.6 Korr Ph 2 M27 (Carl Zeiss) using the inbuilt ‘optimal’ settings to determine the optimal intervals based on sample thickness. Z-projections with the minimum intensity or average intensity setting were obtained using ImageJ/Fiji ver. 1.53t for dermatophyte or *C. albicans* biofilms, respectively.

Dermatophyte biofilm hypha diameters were measured using the “Straight” line function in ImageJ/Fiji ver. 1.53t. A total of 50 hyphae were measured in each condition.

Scanning electron microscopy images were obtained for *Epidermophyton floccosum*. The fungal inoculum was grown on a glass side according to the procedure described in Section 2.2.4. Following essential oil treatments (0.64 and 0.32 µL/mL), glass slides were attached to SEM stubs with adhesive carbon substrates (12 mm, Agar Scientific, Essex, UK) and visualized using a variable-pressure scanning electron microscope (FlexSEM 1000, Hitachi, Tokyo, Japan) at an accelerating voltage of 10 kV.

### 2.3. Statistical Analysis

Results are shown as mean values ± SEM (standard error of the mean) from at least three independent experiments performed in duplicate. Sample normality was determined using the Shapiro–Wilk normality test. Statistical significance for dermatophytes was determined using mixed effect analysis followed by Dunnett’s multiple comparisons test, while for *C. albicans,* it was determined using ordinary one-way analysis of variance (ANOVA) followed by Dunnet’s multiple comparisons test, using GraphPad Prism version 9.5.0 (GraphPad Software, San Diego, CA, USA). Statistical significance is considered for *p* values lower than 0.05.

## 3. Results

### 3.1. Chemical Composition of L. multifida Essential Oil

The essential oil of *L. multifida* obtained from plants growing in Sesimbra region in Portugal was rich in oxygenated monoterpenes, being the phenolic compound carvacrol present in very high amounts (46.4.%). Other main compounds included cis-β-ocimene (12.7%) and β-bisabolene (10.1%), as shown in Table 1.

### 3.2. Effect of L. multifida Essential Oil on the Formation of Dermatophyte Biofilms

Dermatophytes are able to form biofilms that contribute to their higher resistance to antifungal drugs. In our studies, for the majority of the dermatophyte strains tested, two types of fungal growth were observed: aerial and attached. Bearing in mind the general definition of a biofilm, only attached fungi were considered for the evaluation of the effect of *L. multifida* essential oil on both biofilm formation and mature biofilm disruption. In both cases, biofilm mass and extracellular matrix were quantified using crystal violet and safranin staining, respectively, and biofilm viability was assessed using the XTT assay.

**Figure 1:**
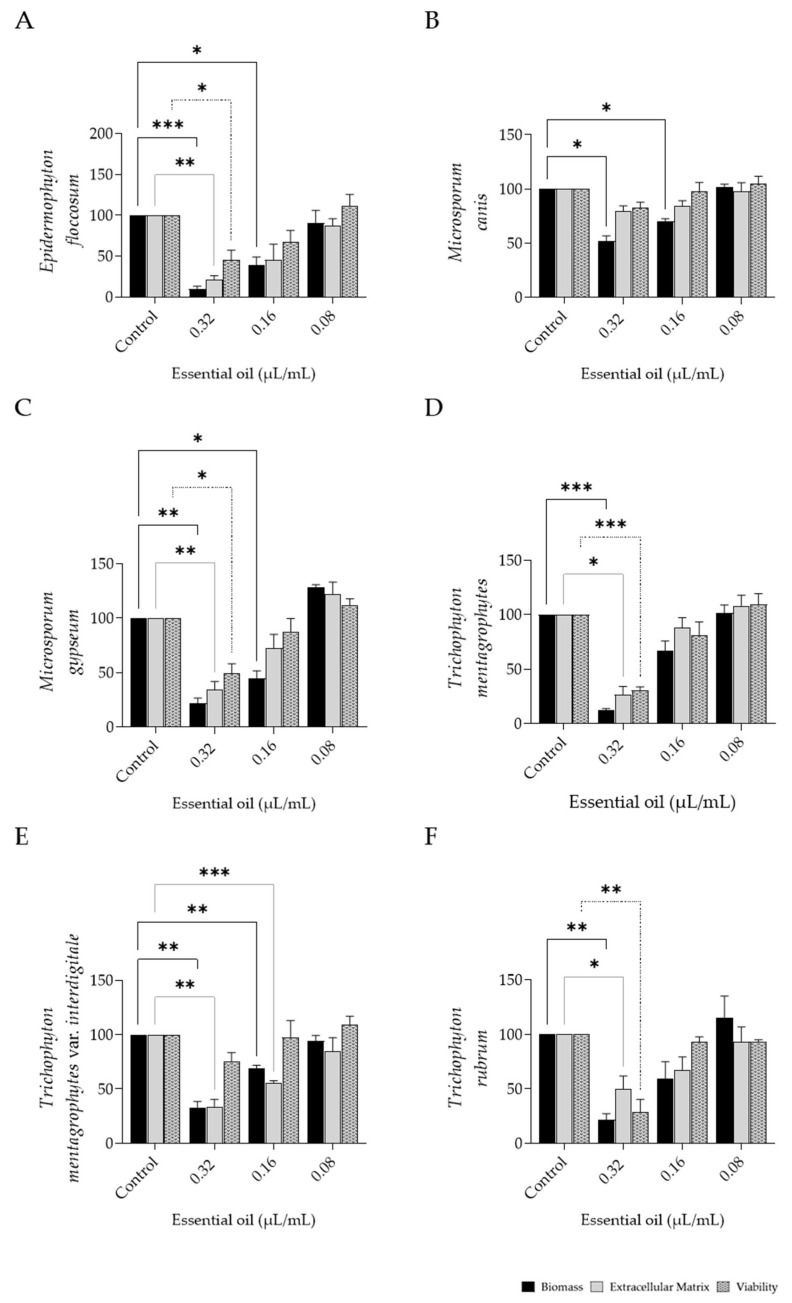
Effect of *L. multifida* essential oil on dermatophyte biofilm formation. Dermatophytes were allowed to adhere to a surface for 3 h and then incubated for 72 h in the presence or absence of the essential oil. The parameters assessed on *Epidermophyton floccosum* (**A**), *Microsporum canis* (**B**), *M. gypseum* (**C**), *Trichophyton mentagrophytes* (**D**), *T. mentagrophytes* var. *interdigitale* (**E**) and *T. rubrum* (**F**) included biofilm mass using crystal violet assay (dark bars), extracellular matrix by safranin staining (gray bars), and biofilm viability using XTT assay (dotted bars). Results are expressed as a percentage relative to the control of a minimum of three independent experiments (mean ± SEM; * *p* < 0.05, ** *p* < 0.01; *** *p* < 0.001, compared to control).

Regarding the effect of the oil on biofilm formation, a significant inhibition was attained at 0.32 µL/mL, with biofilm mass being greatly inhibited in all the tested strains and extracellular matrix and biofilm viability being significantly decreased in the majority of the strains (Figure 1A–F). Combining these features, the essential oil at 0.32 µL/mL was more effective against *E. floccosum*, inhibiting 90% of biofilm mass, 80% of extracellular matrix, and more than 50% of biofilm viability (Figure 1A). In addition, *M. gypseum* (Figure 1C), *T. mentagrophytes* (Figure 1D), and *T. rubrum* (Figure 1F) were quite susceptible, with all parameters reduced by more than 50%. In addition, at 0.16 µL/mL, the essential oil was also effective on dermatophyte biofilm mass, as a significant reduction was observed in several strains (Figure 1A–C,E), confirming its antibiofilm potential.

### 3.3. Effect of L. multifida Essential Oil on the Disruption of Dermatophyte Mature Biofilms

Considering the resistance of mature biofilms to antifungals, we assessed the capacity of *L. multifida* essential oil to disrupt these structures. As expected, the mature biofilms were more resistant to the activity of the essential oil; however, promising results were obtained for all the tested strains, except for *Microsporum cani*s. In addition to the parameters assessed in Section 3.2, morphological alterations were considered, resorting to light microscopy observations and quantification of hypha diameters.

**Figure 2:**
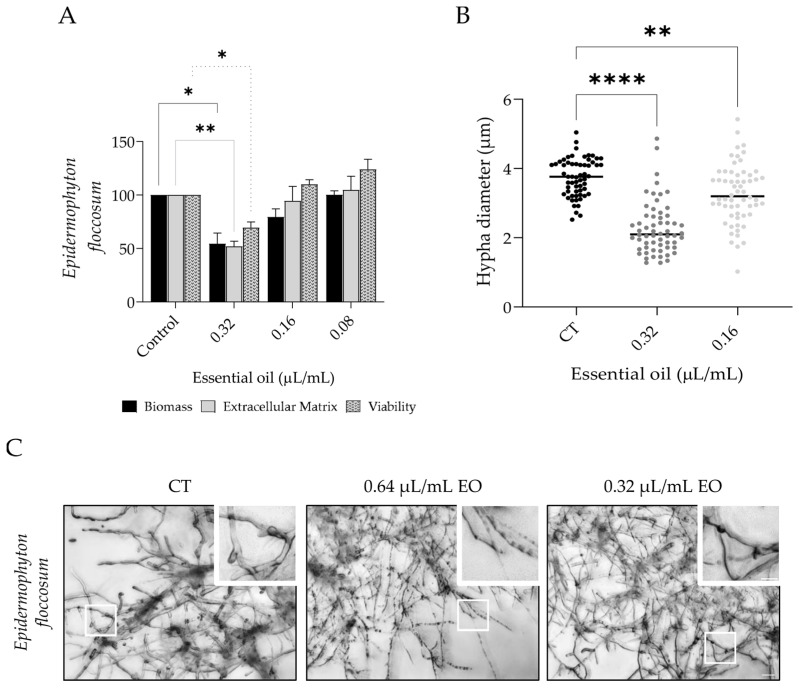
Effect of *L. multifida* essential oil on *Epidermophyton floccosum* biofilm disruption. Dermatophytes adhered for 3 h to a surface and were allowed to grow for 72 h and then exposed to the essential oil for an additional 24 h. The parameters assessed included biofilm mass using crystal violet assay (dark bars), extracellular matrix using safranin staining (gray bars), and biofilm viability using XTT assay (dotted bars) (**A**); hypha diameter (**B**); and optical microscopy observations (**C**). Results are expressed as a percentage relative to the control of a minimum of three independent experiments (mean ± SEM; * *p* < 0.05, ** *p* < 0.01; **** *p* < 0.001, compared to control); scale bar = 20 µm; close-up: 10 µm.

Similar to that observed in biofilm formation inhibition, *E. floccosum* was the most susceptible strain (Figure 2A), with 0.32 µL/mL of the essential oil significantly decreasing biofilm mass (46%), extracellular matrix (49%), and viability (30%). Morphological alterations were also obvious with hyphae being much thinner (Figure 2B) and more septate (Figure 2C).

**Figure 3:**
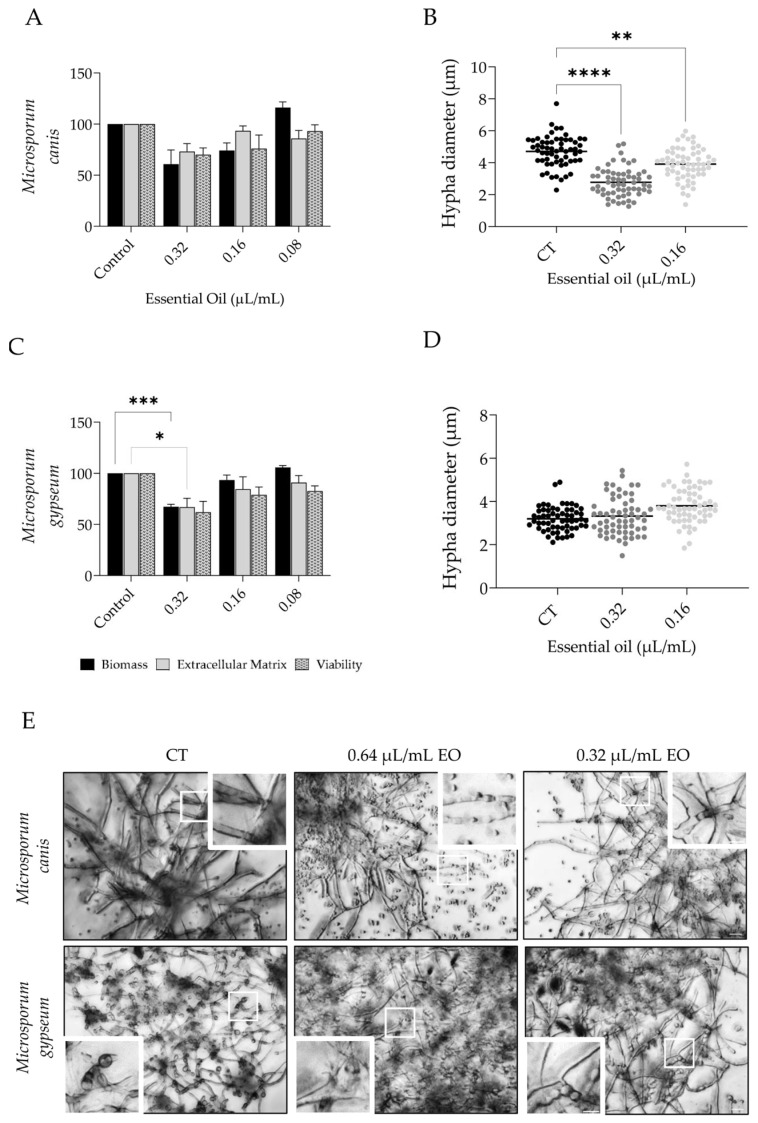
Effect of *L. multifida* essential oil on *Microsporum canis* and *Microsporum gypseum* biofilm disruption. Dermatophytes adhered for 3 h to a surface and were allowed to grow for 72 h and then exposed to the essential oil for an additional 24 h. The parameters assessed included biofilm mass using crystal violet assay (dark bars), extracellular matrix by safranin staining (gray bars), and biofilm viability using XTT assay (dotted bars) (**A**,**C**); hypha diameter (**B**,**D**); and optical microscopy observations (**E**). Results are expressed as a percentage relative to control of a minimum of three independent experiments (mean ± SEM; * *p* < 0.05, ** *p* < 0.01, *** *p* < 0.001, **** *p* < 0.0001, compared to control); scale bar = 20 µm; close-up: 10 µm.

The capacity of the essential oil to disrupt *Microsporum canis* and *M. gypseum* mature biofilms was also assessed (Figure 3). In the first, the oil (0.32 µL/mL) slightly decreased all parameters assessed, although no statistical significance was attained (Figure 3A). This weak activity was also observed using optical microscopy, where the amount of biofilm detected was very similar in all conditions; however, hypha diameter was clearly reduced in the treated biofilms (Figure 3E). Indeed, when hypha diameter was measured, a strong decrease was detected (Figure 3B). On the other hand, for *M. gypseum*, a significant reduction in biofilm mass and extracellular matrix was attained (Figure 3C), and morphological alterations were also observed, with the biofilm presenting a disorganized structure in comparison to the control biofilm (Figure 3E); nevertheless, no effect on hypha diameter was observed (Figure 3D).

**Figure 4:**
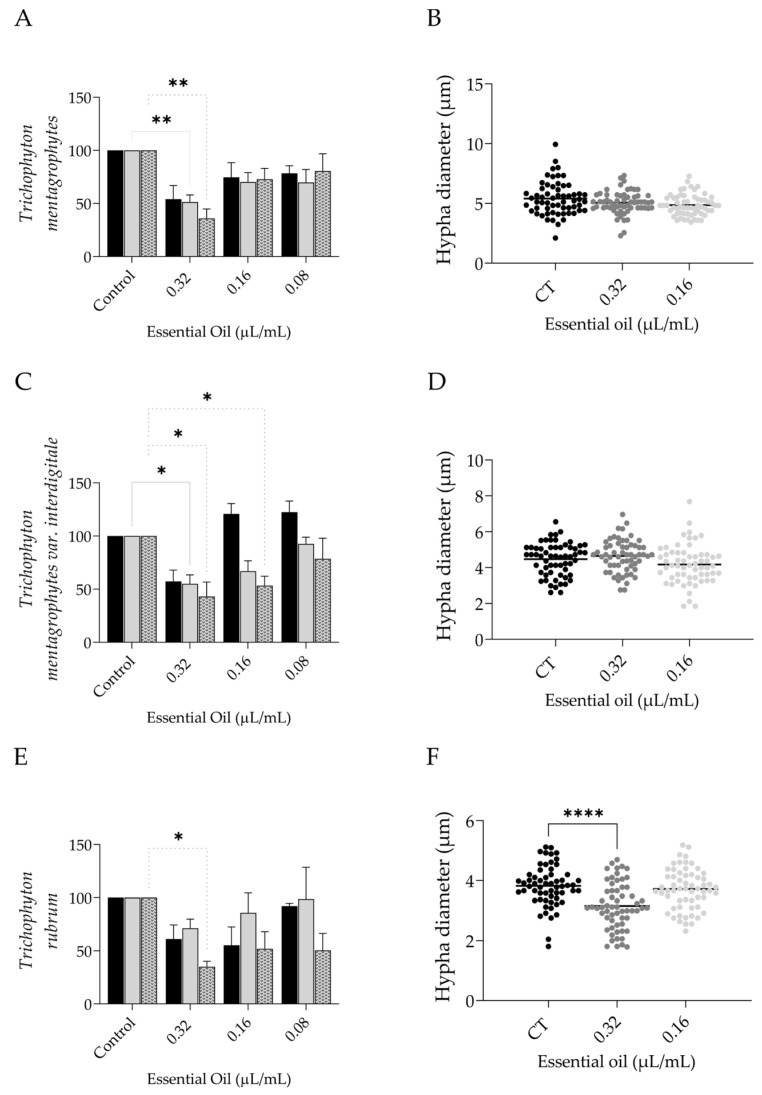

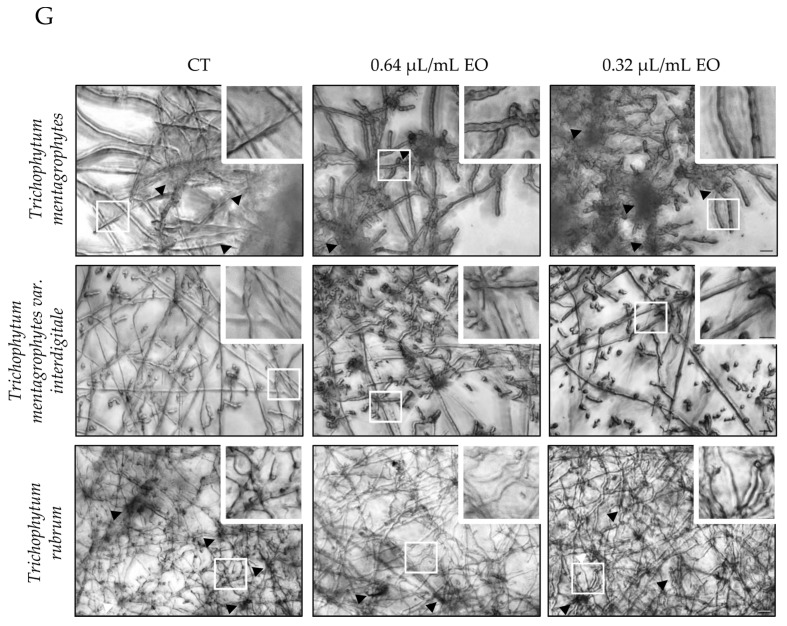
Effect of *L. multifida* essential oil on *Trichophyton mentagrophytes, Trichophyton mentagrophytes* var. *interdigitale,* and *Trichophyton rubrum* biofilm disruption. Dermatophytes adhered for 3 h to a surface and were allowed to grow for 72 h and then exposed to the essential oil for an additional 24 h. The parameters assessed included biofilm mass using crystal violet assay (dark bars), extracellular matrix by safranin staining (gray bars), and biofilm viability using XTT assay (dotted bars) (**A**,**C**,**E**); hypha diameter (**B**,**D**,**F**); and optical microscopy observations (**G**). Results are expressed as a percentage relative to the control of a minimum of three independent experiments (mean ± SEM; * *p* < 0.05, ** *p* < 0.01, **** *p* < 0.0001, compared to control); scale bar = 20 µm; close-up: 10 µm.

For *Trichophyton*, three strains were tested, namely *T. mentagrophytes*, *T. mentagrophytes* var. *interdigitale,* and *T. rubrum* (Figure 4). Overall, the essential oil at 0.32 µL/mL showed a significant reduction in biofilm viability in all the tested strains (Figure 4A,C,E), being also able to significantly reduce extracellular matrix in the first two (Figure 4A,C). Morphological alterations were also observed, particularly for *T. mentagrophytes,* where hyphae appeared bloated, and for *T. mentagrophytes* var. *interdigitale*, where hypha formation seemed compromised (Figure 4G). In agreement with safranin staining, a decrease in the extracellular matrix was observed for *T. mentagrophytes* (Figure 4G, arrowheads). For *T. rubrum,* a reduction in hypha density was observed in the highest dose tested, and, in addition, even though no statistical difference was attained in safranin staining, a reduction in extracellular matrix deposition was observed (Figure 4G, arrowheads). Regarding hypha diameters, significant reductions were only observed in *T. rubrum* (Figure 4F).

### 3.4. Morphological Effect of L. multifida Essential Oil on E. floccosum

**Figure 5:**
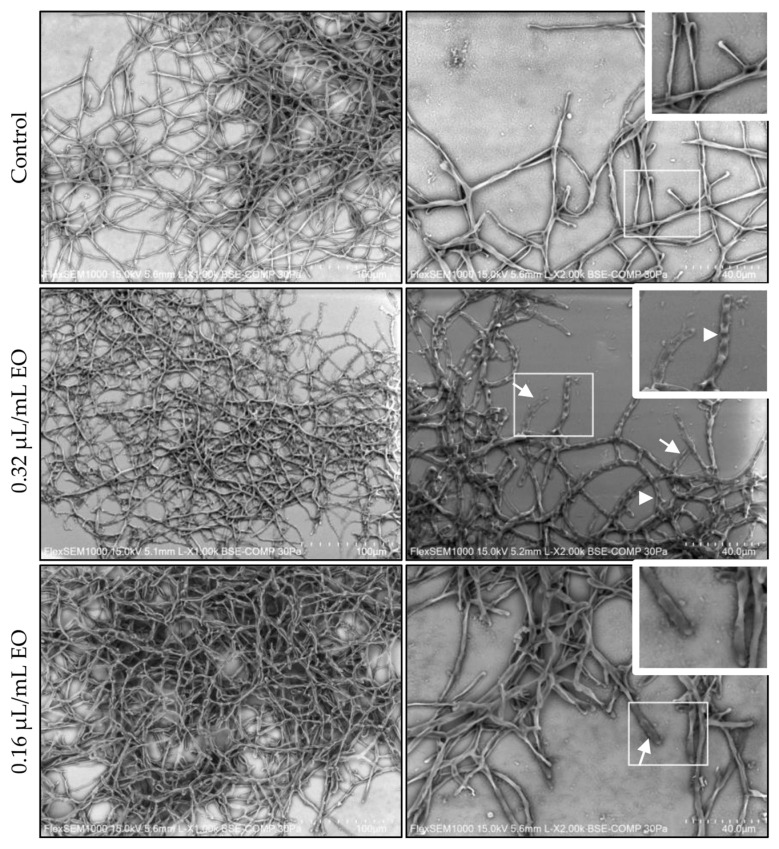
Effect of *L. multifida* essential oil on mature *Epidermophyton floccosum* biofilm morphology. The dermatophyte adhered for 3 h to a glass coverslip and was allowed to grow for 72 h and then exposed to the essential oil for an additional 24 h. Septate (arrowheads) and flattened (arrows) hyphae were observed in the treated fungi, in comparison to the control. Scale bars: 100 µm, close-ups: 40 µm.

*Epidermophyton floccosum* was selected to carry out scanning electron microscopy observations, as it was overall the most susceptible strain to *L. multifida* essential oil. As observed in Figure 5, the essential oil at 0.32 µL/mL induced evident morphological alterations, namely an increase in septate hyphae (arrowhead) with flattened surface (arrows), and less packed mycelia.

### 3.5. Effect of L. multifida Essential Oil on the Formation of Candida albicans Biofilms

Considering the role that *C. albicans* biofilms play in persistent infections, the effect of *L. multifida* essential oil on its biofilm formation and disruption was assessed, through the quantification of biofilm mass and viability.

**Figure 6:**
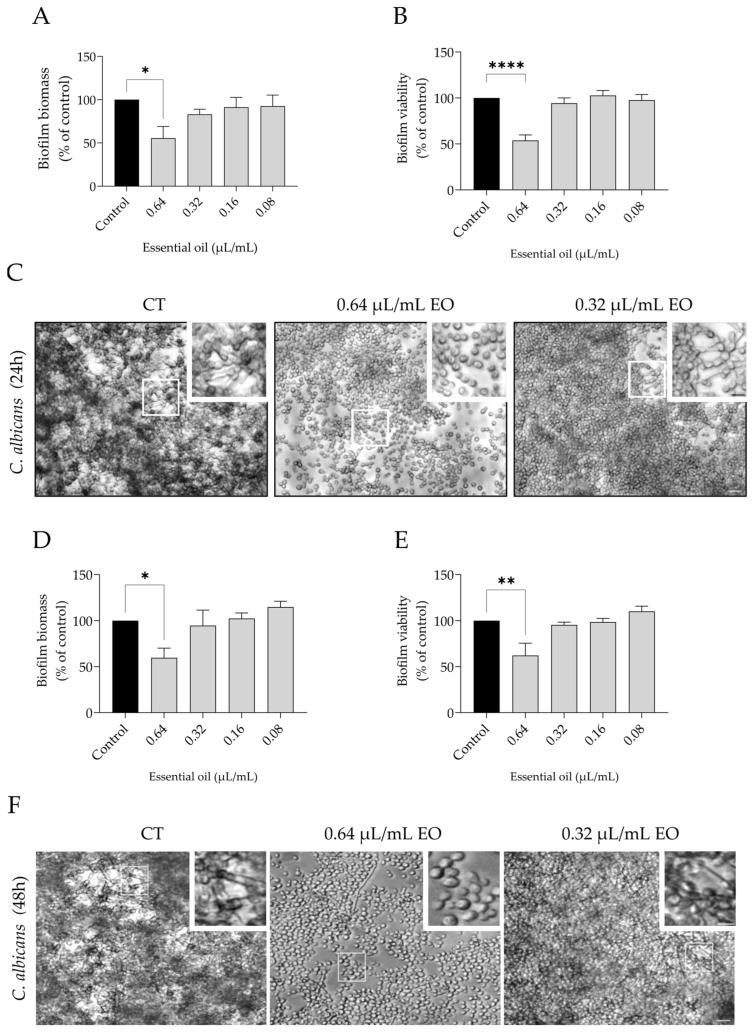
Effect of *L. multifida* essential oil on *C. albicans* biofilm formation. Yeasts were incubated for 24 h (**A**–**C**) or 48 h (**D**–**F**) in the presence or absence of different concentrations of the essential oil. The parameters assessed included biofilm mass using crystal violet assay [(**A**,**D**) for 24 h and 48 h of treatment, respectively]; biofilm viability using XTT assay [(**B**,**E**), for 24 h and 48 h of treatment, respectively]; and morphological alterations through optical microscopy observations [(**C**,**F**) for 24 h and 48 h of treatment, respectively]. Results are expressed as a percentage relative to the control of a minimum of three independent experiments (mean ± SEM, * *p* < 0.05, ** *p* < 0.01, **** *p* < 0.0001, compared to control); scale bar = 20 μm; close-up = 10 µm.

Regarding the effect of the oil on biofilm formation, two time-points were considered: 24 h (Figure 6A–C) and 48 h (Figure 6D–F), thus allowing to assess the effect of the oil on an intermediate (12–30 h) and mature (38–72 h) stage of biofilm development [39].

Overall, the addition of 0.64 µL/mL of the essential oil for 24 h or 48 h was very effective, leading to a reduction in both biofilm mass and viability (Figure 6A,B,D,E). Optical microscopy observations corroborated these results as an evident decrease in biofilms was observed. Indeed, the yeast form of *C. albicans* was more prevalent, indicating a decrease in the dimorphic transition, thus suggesting a reduction in fungal virulence (Figure 6C,F).

### 3.6. Effect of L. multifida Essential Oil on the Disruption of Candida albicans Mature Biofilms

**Figure 7:**
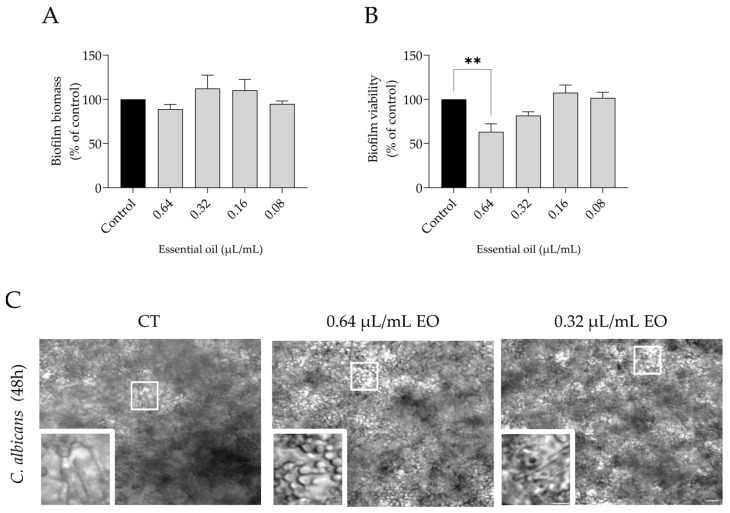
Effect of *L. multifida* essential oil on *C. albicans* mature biofilm disruption. Yeasts were allowed to grow for 24 h and then exposed to the essential oil for 24 h. The parameters assessed included biofilm mass using crystal violet assay (**A**); biofilm viability using XTT assay (**B**) and morphological alterations through optical microscopy observations (**C**). Results are expressed as a percentage relative to the control of a minimum of three independent experiments (mean ± SEM, ** *p* < 0.01, compared to control); scale bar = 20 μm, close-ups = 10 µm.

Mature biofilms, allowed to grow for 24 h, were treated with the essential oil for an additional period of 24 h. As expected, these biofilms were more resilient to the essential oil, with no effects observed on biofilm mass (Figure 7A). Nevertheless, biofilm viability was significantly reduced at 0.64 µL/mL, thus showing a clear effect of the oil before biomass reduction is visible (Figure 7B). Indeed, microscopy observations confirmed similar amounts of biofilm in all conditions, but a closer observation showed quite evident morphological differences (Figure 7C, zoom-ins). Indeed, similar to what was observed during biofilm formation (Figure 6), dimorphic transition was impaired in the presence of 0.64 µL/mL of the essential oil (Figure 7C), reinforcing the effect of the oil on virulence reduction.

## 4. Discussion

Dermatophytosis tends to be neglected as it generally is not life-threatening. However, these infections deserve more attention due to frequent relapses, recalcitrance, and resistance to therapy. Moreover, although predominantly superficial, in certain patients, such as those undergoing immunosuppression therapy, with genetic predisposition, or with chronic pathologies, dermatophytes become invasive, infecting the dermis, and in some cases internal organs such as the brain [40,41]. Their recalcitrance has been associated with long treatment durations that lead to poor patient compliance, host-specific characteristics, the emergence of resistant strains, and the formation of biofilms [8]. Despite the impact of biofilms, primarily on antifungal resistance, studies on dermatophytes are scarce, and the majority report the formation of biofilms in *Trichophyton* species, and only a few assess this capacity in *Microsporum canis* and *M. gypseum*, as reviewed elsewhere [8]. In the present study, besides these strains, we included *Epidermophyton floccosum,* which is also able to produce biofilms in vitro.

Overall, and similarly to that reported by Brilhante and colleagues [9], we observed that *M. canis* was the weakest biofilm producer, as confirmed by optical microscopy analysis (Figure 3) and crystal violet staining (Figure S1). Furthermore, *T. mentagrophytes* var. *interdigitale* also showed a low biofilm formation capacity in comparison to the remaining strains, with *E. floccosum* being the most effective (Figure S1). *C. albicans* was also considered in the present study due to its high prevalence and capacity to form biofilms.

Bearing in mind the need for effective therapeutic strategies able to mitigate the negative impact of dermatophytosis and candidiasis, we sought to explore the antifungal potential of *L. multifida* essential oil rich in carvacrol. The reported chemical composition is in agreement with previous studies using plants from different regions that demonstrate that carvacrol is the predominant compound in this species [27,42,43,44,45,46]. Overall, our results show a significant antibiofilm potential with promising results attained on the prevention of both dermatophyte and *C. albicans* biofilm formation. These results are highly relevant since fungal biofilms are often associated with chronic infections [47]. Besides biofilm formation, the essential oil was also able to disrupt mature biofilms in all the tested strains (except *M. canis*), a feature of uttermost importance, as biofilm disruption increases fungal susceptibility to conventional antifungal drugs [47], and consequently contributes to the eradication of the infection. These results suggest that *L. multifida* essential oil could be considered in clinical practice and contribute to a more effective therapeutic response.

In our studies, *L. multifida* essential oil from 0.32 µL/mL onwards was able to inhibit biofilm formation in all the tested strains, with *E. floccosum* being the most susceptible strain, followed by *T. mentagrophytes*, *M. gypseum,* and *T. rubrum*. As far as we know, this is the first report on the antibiofilm effect against *E. floccosum*, a very relevant opportunist infectious agent in immunocompromised individuals [48]. Moreover, the interesting results obtained against *T. rubrum*, responsible for 70–90% of all dermatophyte infections [48,49], further highlight the potential of *L. multifida* essential oil, as this dermatophyte is responsible for more than 50% of all invasive dermatophytosis [41]. Indeed, in mature *T. rubrum* biofilm, we report a significant viability decrease in the presence of the essential oil before a significant decrease in biofilm mass and matrix was observed, thus suggesting a decrease in fungal virulence capacity.

*C. albicans* biofilm is associated with very high mortality rates, being responsible for fatal infections in up to 50% of adults and 30% of young individuals [12]. Furthermore, these biofilm-related infections have serious economic consequences. Indeed, in the US alone, these infections are associated with an excess of USD 6.5 billion in annual expenditure, often associated with the high resistance to antifungals observed in these structures [14]. In the present study, we showed that *L. multifida* essential oil is able to prevent *C. albicans* biofilm formation and eradicate its mature biofilms. These results are quite relevant, as fluconazole, the most widely used antifungal in the clinic, fails to inhibit biofilms even at doses 200× higher than its minimal inhibitory concentration [50]. The reduction in biofilm biomass during the formation phase suggests that the essential oil prevents the adhesion of yeasts, making them more susceptible to conventional antifungals, thus highlighting once again the essential oil’s relevance in clinical practice. On the other hand, the capacity of the essential oil to decrease the viability of mature biofilms without decreasing biofilm biomass suggests that, similarly to what we suggested for *T. rubrum*, the oil seems to penetrate the biofilm complex structure and compromise the pathogen´s viability and consequent virulence capacity.

Studies reporting the antibiofilm effect of natural products against dermatophytes are scarce, with only one reporting the effect of essential oils and their isolated compounds [34]. Furthermore, regarding *Lavandula* species essential oils, only a few studies have shown their antibiofilm potential against *Candida* spp. For example, the essential oil from *L. dentata* was able to inhibit *C. albicans* biofilm adhesion, proliferation, and viability [51]. The essential oil from *L. stoechas* presented strong antibiofilm activity against collection and clinical strains of *Candida* spp. [52]. The essential oil from *L. x intermedia* presented strong disruptive properties against *C. albicans* mature biofilms [53], and the essential oil of *L. angustifolia* presented relevant antibiofilm activity against several clinical isolates of *C. albicans* [54]. Contrarily to fungal species, studies on bacterial biofilms are more frequent, with several pointing out the antibiofilm potential of lavender species. Overall, *L. angustifolia* essential oil is the most studied and has shown promising disruptive effects against mature *Campylobacter jejuni* biofilms, in addition to preventing its adhesion [55], and against methicillin-resistant *Staphylococcus aureus* (MRSA) biofilms [56]. This oil was also able to eradicate *S. aureus* and *Escherichia coli* mature biofilms from the surface of medical materials [57]. The essential oils from other lavender species have also been studied, such as *L. x intermedia,* which was able to disrupt *Streptococcus agalactiae* mature biofilms [53]; *L. mairei,* which disrupted mature biofilms of *Acinetobacter baumannii*, prevented cell adhesion, and induced cell detachment [58]; and *L. officinalis,* which prevented the adhesion of *Porphyromonas gingivalis* and *Streptococcus mutans* [59].

Regarding the effect of isolated compounds, namely carvacrol, highly present in *L. multifida* essential oil, only one study has been performed on fungi showing its ability to inhibit and disrupt mature biofilms of *Cryptococcus neoformans* and *C. laurentii* [60]. In this study, the possible mechanism of action underlying the observed effect was suggested to be related to its capacity to decrease extracellular matrix and capsule thickness, alter lipid metabolism, inhibit ergosterol synthesis, and induce reactive oxygen species formation [61]. Similar to lavender essential oils, studies on the effect of essential oils´ isolated compounds on bacterial biofilms are quite frequent. Indeed, carvacrol has been widely assessed alone, in combination, or even encapsulated, and has shown effects, namely on biofilm formation inhibition and/or mature biofilm disruption. For example, carvacrol was able to decrease biofilm formation in *Enterococcus faecalis* and *Klebsiella pneumoniae* [62,63], in *Pseudomonas aeruginosa* and *S. aureus* formed on technical surfaces [64], and in *P. gingivalis*, *Fusobacterium nucleatum,* and *S. mutans* biofilm formed on titanium implants [65,66]. In addition, on mature biofilms, this compound was quite effective, being able to eradicate mature biofilms of *Aeromonas hydrophyla* [67], *Staphylococcus epidermidis*, *P. aeruginosa* [68], and *Gardnerella* spp., which, even after compound removal, did not recover their viability. Furthermore, carvacrol prevented the adhesion of *Gardnerella* to human vaginal epithelial cells [69], highlighting its therapeutic potential. Carvacrol was also able to prevent both biofilm formation and mature biofilm disruption in different *Mycobacterium* species [70]. The same effects were reported for *Salmonella* Enteritidis [71], *S. aureus,* and *S. epidermidis* [72]. When microencapsulated, this compound was also quite effective in inhibiting *P. aeruginosa* and *E. faecalis* biofilm formation [73,74]. In addition, carvacrol-loaded chitosan nanoparticles were effective in disrupting mature biofilms of *P. aeruginosa* [75], probably due to alterations in quorum sensing [76]. Treatment with free and encapsulated carvacrol prevented biofilm formation by *Salmonella* and *S. aureus* in stainless steel surfaces [77], while carvacrol-loaded polymeric systems impaired biofilm formation in *S. aureus, S. epidermidis*, *E. coli,* and *Listeria monocytogenes* [78,79]. Furthermore, poly-(DL-lactide-co-glycolide) nanoparticles loaded with carvacrol disrupted mature *S. epidermidis* biofilms [80], and micelles loaded with this compound disrupted mature *E. coli* and *L. monocytogenes* biofilms [81,82]. β-bisabolene, another main compound of *L. multifida* essential oil, was also described as having antibiofilm potential by inhibiting biofilm formation in *Mycobacterium smegmatis* [83]. Therefore, taking into account the reported antibiofilm effects of both carvacrol and β-bisabolene, it seems that the activity herein observed might be due to the presence of these compounds; however, synergistic effects with other compounds cannot be disregarded.

## 5. Conclusions

Herein, we report, for the first time, strong inhibitory effects of *L. multifida* essential oil on biofilm formation against dermatophytes and *C. albicans*. Of relevance, the essential oil was able to eradicate mature biofilms against all tested strains, with dermatophytes being more susceptible than *C. albicans*, particularly *E. floccosum*, *T. mentagrophytes*, *M. gypseum,* and *T. rubrum*. These results are quite relevant, as studies on dermatophytes biofilms are scarce, with this study being the first to consider *E. floccosum* biofilms. The disruptive capacity reported is highly pertinent as it highlights that *L. multifida* essential oil could be of relevance in clinical practice. Indeed, this essential oil has a pleasant aroma, and due to its lipophilicity, can be easily applied to several topical products.

Overall, the present study brings new insights to the field of dermatophytosis and paves the way for the development of complementary strategies to manage mycosis, as these infections are highly prevalent and tend to be neglected. This is quite relevant, as patients with underlying health concerns or a weakened immune system are at higher risk of frequent relapses and recurrent infections. Moreover, in some cases, these fungal infections can evolve into chronic disorders, thus strengthening the need for new therapeutic agents. Nevertheless, in order to validate the promising antibiofilm effects herein reported for *L. multifida* essential oil, further experiments should consider comparisons with reference antifungal drugs, deepen the knowledge on the mechanism of action underlying the antibiofilm effect, and consider pre-clinical studies. In addition, despite *L. multifida*´s wide distribution (Iberian Peninsula, Sicily, Northwest Africa, and the Canary Islands), cultivation techniques should be considered to avoid overexploitation in the wild and enable a standardized chemical composition relevant for industrial purposes.

## Figures and Tables

**Table 1:** Chemical composition of *L. multifida* essential oil.

RI †	RI ‡	Compound	%
931	1029	α-pinene	1.5
961	1441	1-octen-3-ol	1.9
972	1127	β-pinene	0.1
982	1161	myrcene	5.9
1007	1152	3-carene	0.7
1013	1272	ρ-cymene	1.0
1022	1205	limonene	0.5
1027	1233	cis-β-ocimene	12.7
1037	1249	trans-β-ocimene	0.7
1054		octanol	0.6
1067	1401	fenchone	0.2
1073	1435	cymenene	0.7
1079	1285	terpinolene	1.3
1084	1381	nonanal	0.2
1084	1539	linalool	1.5
1099	1577	fenchol	0.2
1118	1370	allo-ocimene	0.3
1170	1689	α-terpineol	0.2
1281	2201	carvacrol	46.4
1411	1591	trans-caryophyllene	1.4
1451	1637	allo-aromadendrene	0.2
1451	1661	trans-β-farnesene	0.2
1469	1699	germacrene D	0.4
1495	1743	(E,E)-α-farnesene	2.3
1498	1720	β-bisabolene	10.1
1510	1748	δ-cadinene	0.3
1556	2107	spathulenol	1.7
1560	1966	caryophyllene-oxide	1.0
1619	2170	T-muurolol	0.3
1632	2216	α-cadinol	0.3
1662	2211	α-bisabolol	0.4
Monoterpene hydrocarbons			25.1
Oxygen-containing monoterpenes			48.8
Sesquiterpene hydrocarbons			14.9
Oxygen-containing sesquiterpenes			3.7
Others			2.7
TOTAL			95.2

Compounds listed in order of their elution on the SPB-1 column. † Experimental retention indices on the SPB-1 column relative to C8–C24 n-alkanes. ‡ Experimental retention indices on the SupelcoWax-10 column relative to C8 to C24 n-alkanes.

## Data Availability

The data presented in this study are available on request from the corresponding author.

## Supplementary Materials

The following supporting information can be downloaded at: https://www.mdpi.com/article/10.3390/pharmaceutics15082142/s1, Figure S1: Dermatophytes biofilm biomass assessed by crystal violet assay. Dermatophytes were left to adhere for 3 h and further incubated for 72 h in culture medium. Values represent the mean ± SEM of the optical density at 620 nm of at least three independent assays.
