# Supplementary material for: Antibiofilm Effect of Lavandula multifida Essential Oil: A New Approach for Chronic Infections
Source: Pharmaceutics. 2023 Aug 15;15(8):2142. doi: 10.3390/pharmaceutics15082142 (PMC10458520; doi:10.3390/pharmaceutics15082142)
Supplement: Supplementary file 1 [file pharmaceutics-15-02142-s001.zip › pharmaceutics-2485221-supplementary.pdf]

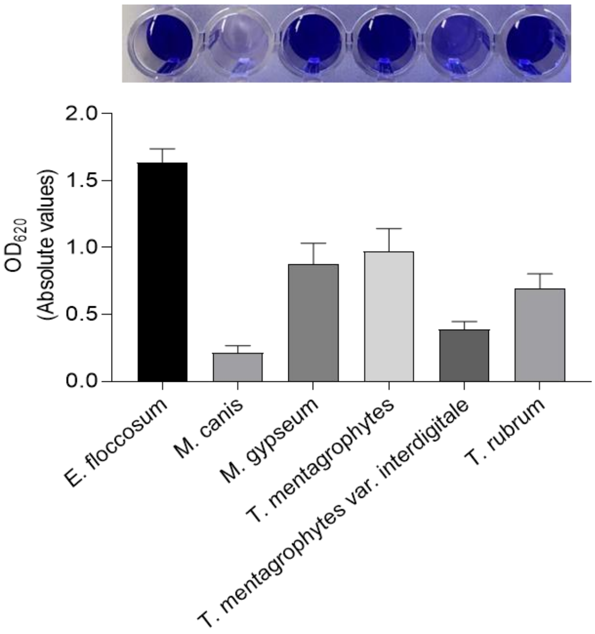

Figure S1. Dermatophytes biofilm biomass assessed by crystal violet assay. Dermatophytes were left to adhere for 3h and further incubated for 72h in culture medium. Values represent the mean ± SEM of the optical density at 620 nm of at least three independent assays.
